# Enhancing ST-Elevation Myocardial Infarction Diagnosis and Management: The Integral Role of Echocardiography in Patients Rushed to the Cardiac Catheterization Laboratory

**DOI:** 10.3390/jcm13051425

**Published:** 2024-02-29

**Authors:** Gemma Marrazzo, Stefano Palermi, Fabio Pastore, Massimo Ragni, Alfredo Mauriello, Aniello Zambrano, Gaetano Quaranta, Andrea Manto, Antonello D’Andrea

**Affiliations:** 1 Department of Cardiology, Umberto I° Hospital, 84014 Salerno, Italy; gemmamarrazzo@gmail.com (G.M.); fabio.pastore1981@gmail.com (F.P.); massimo.ragni@gmail.com (M.R.); gaetano.quaranta67@gmail.com (G.Q.); 2 Public Health Department, University of Naples Federico II, 80131 Naples, Italy; stefano.palermi@unina.it; 3 Department of Cardiology, Luigi Vanvitelli University, 81100 Naples, Italy; alfredo.mauriello93@libero.it; 4 Department of Neuroradiology, Umberto I° Hospital, 84014 Salerno, Italy; andrea.manto@gmail.com

**Keywords:** echocardiography, myocardial infarction, point-of-care ultrasound

## Abstract

Coronary artery disease (CAD) remains a significant global health concern, necessitating timely and precise diagnosis, especially for acute coronary syndromes (ACSs). Traditional diagnostic methods like electrocardiograms (ECGs) are critical, yet the advent of echocardiography has revolutionized cardiac care by providing comprehensive insights into heart function. This article examines the integration of echocardiography in the cardiac catheterization laboratory, emphasizing its role in augmenting traditional diagnostics, enhancing patient outcomes, and preparing for targeted interventions. Specifically, we argue for the routine use of focused echocardiographic evaluations in patients presenting with ST-Elevation Myocardial Infarction (STEMI) to the cath lab, illustrating how this practice can significantly refine diagnostic accuracy, identify concurrent life-threatening conditions, and inform the management of STEMI and its complications.

## 1. Introduction

Coronary artery disease (CAD) stands as a principal contributor to global morbidity and mortality, demanding swift and precise diagnosis, particularly for acute manifestations like acute coronary syndromes (ACSs). Although electrocardiograms (ECGs) have been fundamental in ACS diagnosis, the advent of echocardiography has markedly revolutionized cardiac care. This non-invasive, versatile diagnostic modality enhances patient management by offering comprehensive cardiac assessments. The integration of echocardiography into the cardiac catheterization laboratory setting signifies a paradigm shift, marrying traditional diagnostic approaches with cutting-edge imaging techniques to improve patient outcomes. This evolution from a basic diagnostic tool to a cornerstone of interventional cardiology is propelled by technological advancements, enriching the clinician’s arsenal for precise diagnosis and effective intervention in ACS management. Through this article, we explore the symbiotic relationship between echocardiography and cardiac catheterization, highlighting its impact on refining diagnostic accuracy, facilitating targeted interventions, and shaping the future of cardiac care. 

## 2. The Role of Echocardiography in the Diagnosis of CAD

Myocardial ischemia and infarction, as primary manifestations of CAD, exhibit a spectrum of clinical presentations that necessitate prompt and accurate diagnostic approaches [1]. Echocardiography has emerged as a crucial tool, offering insights beyond the capabilities of traditional ECGs, particularly in emergency situations [2,3]. This technique allows for the assessment of myocardial systolic function, both globally and segmentally; evaluates valvular integrity; and identifies other structural heart diseases, providing a comprehensive evaluation in acute settings (Table 1 and Table 2). The advent of Focused Cardiac Ultrasound (FoCUS) represents a paradigm shift toward rapid, bedside cardiac assessments, essential in the fast-paced environment of emergency care [4]. In the cardiac catheterization laboratory, echocardiography brings significant benefits such as its non-invasive nature, lack of ionizing radiation, and the convenience of bedside use, ensuring patient safety and allowing for repeated assessments. Specifically, focused transthoracic echocardiography (TTE) is utilized, employing apical and subxiphoid views in a two-dimensional mode for qualitative or semi-quantitative assessments, offering clear answers to direct clinical questions [5]. Yet, it is imperative to balance the use of echocardiography to avoid delaying critical interventions, such as revascularization in ST-Elevation Myocardial Infarction (STEMI) cases. TEE is selectively used in emergencies for differentiating between acute coronary and aortic syndromes or diagnosing endocarditis with suspected valvular perforation. The use of Doppler vascular ultrasound for precise access to the common femoral artery during interventions, especially in scenarios of cardiogenic shock, highlights its critical role in supporting procedural success and optimizing patient outcomes. The expanded application of echocardiography, along with a detailed understanding of its capabilities and limitations, emphasizes its vital role in contemporary cardiac emergency protocols, advocating for its systematic integration into diagnostic and treatment strategies. This expansion of echocardiography’s role underscores its indispensable contribution to modern cardiac care, particularly in the emergency and interventional settings, where its accuracy, speed, and non-invasiveness significantly impact patient management and outcomes.

## 3. The Optimal Management of Patients Rushed to the Cardiac Catheterization Laboratory in Emergencies

In patients with ST-Elevation Myocardial Infarction (STEMI), the urgency for reperfusion can sometimes overshadow a comprehensive diagnosis, potentially leading to interventions without fully considering non-coronary causes of symptoms [6]. For patients at non-primary percutaneous coronary intervention (PCI) centers with suspected STEMI, the “door-in to door-out” time is a critical clinical performance measure, recommended to be 30 min or less to expedite reperfusion therapy [7]. The 2017 guidelines introduced the “hub and spoke” model for STEMI networks, aiming to ensure timely primary PCI for more patients and to improve clinical outcomes [6]. Emergency Medical Services (EMSs) are advised to transport patients with a presumptive diagnosis of STEMI directly to hospitals offering 24/7 PCI services, bypassing hospitals without PCI capabilities, based solely on ECG diagnosis [8].

Typically, patients rushed to the cardiac catheterization laboratory in emergencies are diagnosed with STEMI or its equivalent, meaning they undergo invasive coronary angiography and intervention based primarily on symptoms (such as chest pain, dyspnea, syncope, or general malaise) and the results of a 12-lead ECG. For these reasons, in the emergency management of STEMI, swiftly performing an echocardiographic scan that concentrates on acquiring key elements can be crucial for the appropriate management of acute patients in the catheterization laboratory. Clinical studies are highly recommended to determine whether this approach has advantages or disadvantages in this patient category.

Current guidelines do not advocate routine echocardiography in STEMI patients unless the diagnosis is uncertain or the patient is hemodynamically unstable [7]. This is to prevent potential delays in artery revascularization. However, recent developments in computerized diagnostic algorithms for ECG interpretation, especially useful in pre-hospital settings, have shown promise [9]. These computerized measurements offer instant, unbiased results and can measure waveforms with high precision [10]. When STEMI is suggested, paramedics can take specific actions to optimize patient care, including activating a cardiac catheterization laboratory and directing patients to PCI-compatible STEMI centers. Despite these advancements, the sensitivity and specificity of pre-hospital computer algorithm interpretations for identifying STEMI are still under debate [11]. A study comparing different ECG interpretation algorithms found no significant differences in sensitivity and specificity among them [12]. Another study highlighted significant disagreement among physicians in interpreting ECGs with STEMI characteristics, questioning their suitability as standalone diagnostic tests for STEMI. Conversely, performing a “focused” echocardiographic scan on all patients arriving to the cath lab for STEMI could greatly enhance the sensitivity and specificity of the diagnosis, identify alternative life-threatening pathologies requiring different rapid interventions, and better frame the type of STEMI and associated complications, preparing for the most appropriate intervention [13]. Liu et al. demonstrated that cases of acute aortic dissection with ST-Elevation Acute Coronary Syndrome (STE-ACS) resulted in significantly lower mortality compared to cases where such examination was delayed, leading to unnecessary and potentially harmful treatments [14]. A study involving 385 patients presenting to the emergency department for chest pain evaluated the sensitivity and specificity of the method, concluding that bedside echocardiography had high specificity (90.4%) and sensitivity (97.43%) in identifying regional wall motion abnormality due to ACSs [15]. The European Society of Cardiology has recommended emergency echocardiography competence for various acute pathologies, highlighting its fundamental role in emergency settings [6]. Thus, in the emergency management of STEMI, a rapid echocardiographic scan, focused on acquiring key elements, could have a viable role in accurate patient management in the cath lab. Clinical studies are needed to determine whether this approach offers advantages or disadvantages in this patient category. In emergency rooms and chest pain units, transthoracic TTE should be routinely available, performed, or interpreted by trained professionals [6]. The ESC 2023 guidelines confirm a secondary role of the echocardiogram during STEMI, carving out a role only in the case of hemodynamic instability, suspected mechanical complications, STEMI-complicated cardiogenic shock, or unclear STEMI diagnosis (TTE can be useful to identify signs suggestive of ongoing ischemia or prior MI) (Table 3) [6]. Indeed, guidelines do not recommend routine echocardiography in patients with STEMI unless the diagnosis is doubtful or the patient has hemodynamic instability (Table 4). This is to avoid potential delays in revascularizing the artery responsible for AMI. The high sensitivity and specificity of the ECG in diagnosing STEMI are presupposed to avoid unnecessary and potentially dangerous invasive examinations or delaying the diagnosis of an alternative pathology to STEMI due to symptoms and ECG changes.

Recent developments in computerized diagnostic algorithms for ECG interpretation, particularly useful in pre-hospital environments, have assisted ECG interpretations [16]. Computer ECG measurements are instantaneous, eliminate human bias, and can accurately measure waveforms with high resolution. When STEMI is suggested, paramedics perform specific actions to optimize patient care, including activating a cardiac catheterization laboratory and routing patients directly to PCI-compatible STEMI centers [17]. However, the sensitivity and specificity of pre-hospital computer algorithm interpretations for identifying STEMI are not very high, and there is significant disagreement among physicians in interpreting ECGs with STEMI characteristics. This questions their suitability as standalone diagnostic tests for STEMI. Conversely, performing a “focused” echocardiographic scan on all patients arriving in the cath lab for STEMI could significantly increase the sensitivity and specificity of the diagnosis, diagnose alternative life-threatening pathologies, and better frame the type of STEMI and any associated complications, preparing for the most appropriate intervention. The integration of early echocardiography presents a crucial advancement, offering a nuanced approach to diagnosis and treatment [18,19]. This method not only confirms STEMI but also uncovers other critical conditions mimicking similar symptoms. The utility of echocardiography lies in its ability to identify regional wall motion abnormalities, assess myocardial wall thickening, and evaluate hemodynamic status, thus providing a detailed assessment surpassing ECGs’ reach. However, practical application necessitates acknowledging its limitations and the importance of timely intervention. To facilitate clinical practice, clear indications for echocardiography before reperfusion include (1) suspected STEMI with atypical ECG findings; (2) hemodynamic instability without a clear diagnosis; and (3) evaluation for mechanical complications of myocardial infarction. The advantages of echocardiography in this clinical setting are yet to be demonstrated against a potential lengthening of the revascularization time. For these reasons, in the emergency management of STEMI, the rapid execution of an echocardiographic scan, focused on acquiring few decisive elements, may be essential for the correct management of acute patients in the cath-lab. Clinical studies focused on answering the question of whether there is an advantage or a disadvantage in this category of patients would be highly recommended.

## 4. Rationale for the Use of Echocardiography for the Diagnosis of STEMI

**Figure 1:**
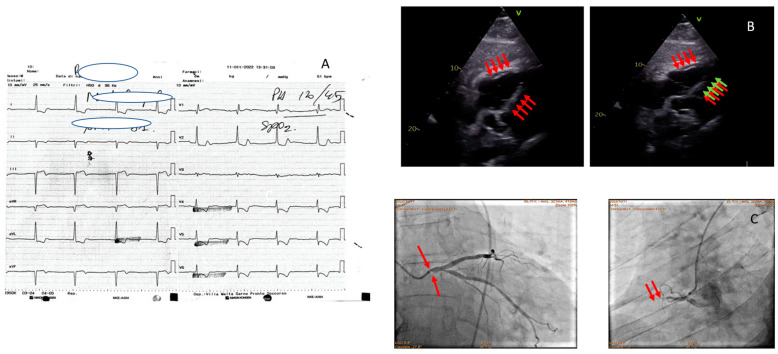
An 81-year-old man with acute chest pain and hypotension. ECG showing diffuse ST depression (**A**). Echocardiogram: subcostal and apical sections show dilatation and akinesia of the free wall of the RV (red and green arrows) (**B**). Coronary angiography; left oblique projection shows non-dominant Cdx closed to the proximal tract (red arrows, on the left picture); left caudal oblique projection shows critical left main ostial stenosis (red arrows, on the right picture), Cx branch free from critical lesions, IVA free from critical lesions (**C**).

Echocardiographic evaluation in STEMI is pivotal for identifying changes in left ventricle (LV) or right ventricle (RV) regional kinetics (hypokinesia, akinesia, dyskinesia) resulting from epicardial coronary artery occlusion, leading to transmural myocardial ischemia [17] (Figure 1). It is essential to assess these kinetics alongside myocardial wall thickening. When acute ischemia affects more than 20% of the parietal thickness, it compromises the systolic thickening of the myocardial wall, even if wall kinetics may be less altered. During STEMI, a resting echocardiogram can reveal both alterations in regional kinetics and a reduction in systolic thickening, with the latter being particularly sensitive in diagnosing myocardial ischemia [21]. Moreover, there is a correlation between the area of regional asynergy and the occluded coronary artery. Kinetic alterations can precede ECG changes and even pain, making them sensitive and early markers of myocardial ischemia. However, the resting echocardiogram is highly sensitive but less specific, as alterations in regional kinetics may not be exclusive to myocardial ischemia [12].

In clinical practice, a rapid echocardiographic confirmation in patients with an ECG diagnosis of STEMI, conducted while preparing for emergency coronary angiography, could complement the invasive procedure. Specifically, it has the following benefits:

1. Effective in confirming or refuting an electrocardiographic-only diagnosis of STEMI;
2. Capable of guiding interventional cardiologists in customizing the revascularization procedure for each patient;
3. Useful in stratifying patients with STEMI by identifying categories that require more aggressive treatments or additional pharmacological support beyond the revascularization procedure alone.

The echocardiogram, in addition to swiftly confirming the diagnosis of STEMI in the cath lab, can also guide the revascularization procedure. Pre-procedure echocardiography identified an RV infarction, influencing the decision-making process in a high-risk procedure.

**Figure 2:**
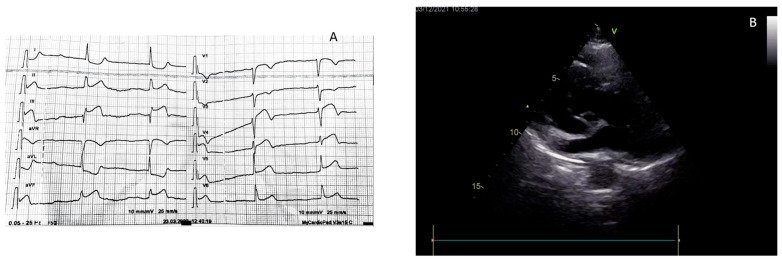
A 78-year woman with chest pain and dyspnea. The ECG showed ST elevation in D2, D3, and AVF leads (**A**). Before access to the cath lab, echocardiography in the emergency room showed severe aortic root dilatation with an evident intimal flap (**B**). The patient as a consequence was not rushed into the cath lab but was sent to cardiac surgery department in the emergency.

**Figure 3:**
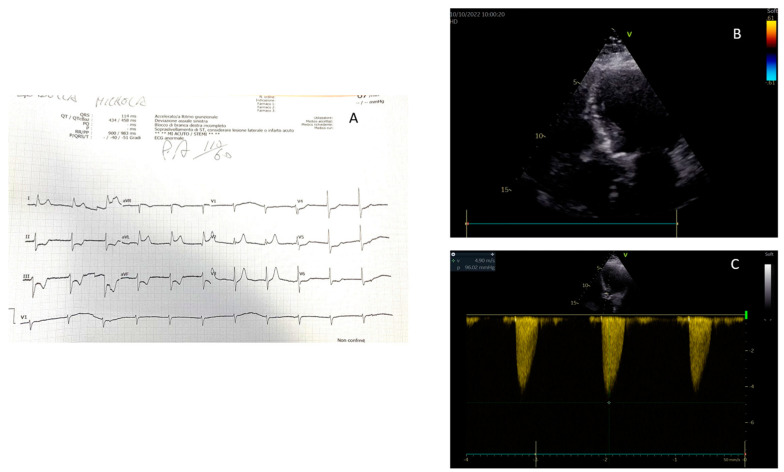
A 78-year woman with chest pain and dyspnea. The ECG showed ST elevation in D1 and AVL leads (**A**) Before access to the cath lab, echocardiography in the emergency room showed normal regional and global left ventricular function (**B**) and a severe aortic stenosis (**C**). The subsequent coronary angiography did not show any critical lesion.

The frequency of false-positive activations in cardiac catheterization laboratories for suspected STEMI is notable in community practice (Figure 2 and Figure 3). Performing an echocardiogram in the cath lab can assist in refining the diagnosis. Additionally, not all coronary occlusions correspond to ST elevation on the ECG. Sometimes, patients requiring urgent revascularization with acute coronary artery occlusion may present with chest pain without an ST elevation pattern, potentially leading to classification as NSTEMI. These electrocardiographically occult occlusive myocardial infarctions (OMIs) are associated with significant delays in catheterization and reperfusion, as well as high morbidity. Conducting an echocardiographic examination in the cath lab for patients with typical chest pain without clear ST elevation on the ECG can help detect regional wall motion abnormalities, aiding in the identification of patients with OMIs [22].

Echocardiography in diagnosing STEMI also facilitates the detection of complications such as ventricular septal rupture or papillary muscle rupture, which are critical for immediate management and surgical intervention [23]. Additionally, it can assess the effectiveness of reperfusion therapy by observing the improvement in myocardial function post-intervention, offering real-time feedback on treatment efficacy. Moreover, echocardiography can play a significant role in the long-term monitoring of patients post-STEMI, evaluating left ventricular remodeling and guiding ongoing therapeutic decisions to prevent heart failure and improve survival rates [24].

## 5. Rationale for the Use of Echocardiography for the Management of STEMI

Mechanical complications of myocardial infarction (MI) are categorized into early and late phases, reflecting the timing post-infarction [25] (Table 5). The widespread adoption of early percutaneous revascularization in acute myocardial infarction (AMI) patients over the last two decades has positively influenced the incidence rates of these complications. However, delayed access to emergency services can increase the likelihood of encountering such complications, often identified in patients who exhibit hemodynamic instability without typical chest pain symptoms, presenting instead with signs of hypoperfusion and systemic or pulmonary congestion [26]. In these scenarios, bedside echocardiography becomes indispensable, providing a rapid and accurate assessment of potential complications necessitating immediate cardiac surgery. This diagnostic tool is key in minimizing delays in both diagnosis and treatment, significantly enhancing patient outcomes in situations traditionally associated with high mortality rates. Echocardiography’s ability to swiftly identify mechanical complications, such as ventricular septal defects, papillary muscle rupture, or free wall rupture, underscores its critical role in the acute management of MI patients, ensuring that timely and appropriate interventions are implemented.

## 6. Conclusions

The integration of echocardiography into the emergency management of STEMI represents a major leap forward in cardiac care, enhancing diagnostic precision and enabling a deeper understanding of cardiac conditions beyond what ECGs can reveal. Echocardiography plays a crucial role in identifying wall motion abnormalities and providing hemodynamic data, critical for diagnosing STEMI and differentiating it from other serious cardiac issues. Despite its significant benefits, challenges such as operator dependency and interpretation variability remain. Future research should aim at addressing these limitations through standardized protocols and training. The exploration of cutting-edge technologies, including pre-hospital echocardiography by EMS, tele-echocardiography, and the incorporation of artificial intelligence for instantaneous interpretation, promises to further transform the management of acute coronary syndromes. Clinical studies are needed to affirm the impact of routine echocardiography in STEMI care and investigate these innovative methods, paving the way for the next advancements in cardiac emergency treatment.

## Figures and Tables

**Table 1:** Functions of FoCUS echocardiography in emergency settings.

Confirm diagnosis of ACS excluding other causes of ST-segment elevation and other forms of acute non-coronary chest pain
Evaluate global and RWMAs
Evaluate wall thickness and search for any pre-existing scars
Study of diastolic function and any pre-existing valve disease to guide supportive pharmacological therapy during PCI
Exclude or confirm RV involvement
Study of the pericardium
Study of mechanical complications such as acute mitral insufficiency (degree and mechanisms), rupture of the interventricular septum or the free wall of the left ventricle, or ventricular thrombosis
Provide important prognostic information that can guide therapeutic strategies in the short and long term

ACS: acute coronary syndrome; RWMAs: regional wall motion abnormalities; PCI: percutaneous coronary intervention; RV: right ventricle.

**Table 2:** Echocardiographic findings of chest pain-related diseases.

Pathology	Diagnostic Sign on Echocardiography
Acute coronary syndrome	Regional wall motion abnormalities, presence of any mechanical complications, intravascular volume assessment, circulatory compromise/shock, cardiac arrest
Acute aortic syndrome	Intimal flap, acute AI, aortic dilatation
Acute myocarditis	RWMAs
Pericardial effusion	Anechoic/hypo-echoic pericardial free space
Tamponade	Pericardial effusion, signs of compression (collapse: RA systolic; RV diastolic; LA systolic; LV diastolic)
Acute pulmonary embolism	Right heart dilation, increased TR velocity, diastolic movement of the IVS, McConnell sign, dilated IVC
Aortic stenosis	Thickened leaflets, gradient
Hypertrophic cardiomyopathy	Myocardial hypertrophy, SAM
Chest trauma	Pericardial/pleural effusion, RWMAs, acute valve insufficiency, acute aortic syndrome

AI: aortic insufficiency; RWMAs: regional wall motion abnormalities; TR: tricuspid regurgitation; IVS: interventricular septum; IVC: inferior vena cava; SAM: systolic anterior motion.

**Table 3:** List of acute cardiovascular conditions in which “emergency echocardiography” may be useful.

ACS/Acute Myocardial Infarction	Global and RWMAs
Mechanical complications of acute myocardial infarction	Mitral insufficiency for rupture of papillary muscle; acute/subacute rupture of free ventricular wall; rupture of interventricular septum; early post-infarction pericarditis; LV aneurysm; LV thrombus
Prosthetic valve dysfunction	Pannus or thrombosis; dehiscence; endocarditis
Heart valve diseases	Stenosis or insufficiency
Pericarditis/Cardiac Tamponade	Anechoic/hypo-echoic pericardial free space; pericardial effusion; signs of compression (collapse: RA systolic; RV diastolic; LA systolic; LV diastolic)
Cardiomyopathies/Myocarditis/Takotsubo	Global and regional wall motion abnormalities; parietal thickness; diastolic function; valve diseases; atrial dilatation
LVAD malfunction	Thrombosis; endocarditis; malposition
Acute aortic syndrome/aortic dissection	Intimal flap; acute AI; aortic dilatation
Acute pulmonary embolism	Signs of acute overload of the right ventricle and pulmonary hypertension
Acute heart failure/cardiogenic shock	Stroke volume; cardiac output; inferior vein cava
Acute complications in the cath lab	Pericardial effusion; cardiac tamponade; heart rupture
Acute complications of cardiac surgery	PNX; pericardial effusion; cardiac tamponade; valvular lesions
Endocarditis	Vegetations: abscesses; pseudoaneurysm; dehiscence; fistulae;
Tumors	
PNX	Barcode sign; lung pulse and sliding sign absence; line A absence
Traumatic injuries of the heart	Pericardial effusion; cardiac tamponade; heart rupture

ACS: acute coronary syndrome; RWMAs: regional wall motion abnormalities; LV: left ventricle; LVAD: left ventricular assist device; AI: aortic insufficiency; PNX: pneumothorax.

**Table 4:** Recommendation of latest cardiological guidelines about the use of echocardiography in emergencies.

ESC 2023 [6]		AHA 2021 [20]	
Recommendations	Class and Level		Class of Recommendations (COR) and Level of Evidence (LOE)
Emergency TTE is recommended in patients with suspected ACS presenting with cardiogenic shock or suspected mechanical complications.	I C	For intermediate-risk patients with acute chest pain, TTE is recommended as a rapid, bedside test to establish baseline ventricular and valvular function, evaluate for wall motion abnormalities, and assess for pericardial effusion.	1 C-EO
Emergency TTE should be considered at triage in cases of diagnostic uncertainty but this should not result in delays in transfer to the cardiac catheterization laboratory if there is suspicion of an acute coronary artery occlusion.	IIa C	In patients with acute chest pain in whom other potentially life-threatening nonischemic cardiac conditions are suspected (e.g., aortic pathology, pericardial effusion, endocarditis), TTE is recommended for diagnosis.	1 C-EO
Immediate echocardiographic assessment is indicated when mechanical complications are suspected.	I C	In patients with acute chest pain and suspected myopericarditis, TTE is effective in determining the presence of ventricular wall motion abnormalities, pericardial effusion, valvular abnormalities, or restrictive physiology.	1 C-EO
		In patients presenting with acute chest pain with a suspected or known history of VHD, TTE is useful in determining the presence, severity, and cause of VHD.	1 C-EO
		In patients presenting with acute chest pain with suspected or known VHD in whom TTE diagnostic quality is inadequate, TEE (with 3D imaging if available) is useful in determining the severity and cause of VHD.	1 C-EO

TTE: trans thoracic echocardiography; ACS: acute coronary syndrome; VHD: valvular heart disease.

**Table 5:** Mechanical complications of myocardial infarction.

Papillary muscle rupture and acute mitral regurgitation
Ventricular septal defect
Rupture of the ventricular free wall
Pseudoaneurysm
Left ventricular apex thrombosis

## Data Availability

Data are available, upon reasonable request, to the corresponding author.
